# Understanding the intersections between ethnicity, area-level deprivation, and inpatient-related features amongst patients with psychotic disorders: a mental health electronic records analysis

**DOI:** 10.1007/s00127-025-02908-1

**Published:** 2025-05-05

**Authors:** Charlotte Humphreys, Jo Hodgekins, Hitesh Shetty, Peter Schofield, Rob Stewart, Sherifat Oduola

**Affiliations:** 1 Department of Clinical Psychology and Psychological Therapies, Norwich Medical School, University of East Anglia, Norwich, NR4 7TJ UK; 2 Cambridgeshire and Peterborough NHS Foundation Trust, Elizabeth House, Cambridgeshire, UK; 3 South London & Maudsley NHS Foundation Trust, Denmark Hill, London, SE5 8AZ UK; 4 School of Life Course and Population Sciences, Faculty of Life Sciences & Medicine, King’s College London, London, UK; 5 Department of Psychological Medicine, Institute of Psychiatry, Psychology & Neuroscience, King’s College London, London, UK; 6 School of Health Sciences, University of East Anglia, Norwich Research Park, Norwich, NR4 7TJ UK

**Keywords:** Psychotic disorder, Deprivation, Ethnicity, Inpatient, Compulsory admission, Intersectionality

## Abstract

**Purpose:**

Ethnic and area-level deprivation disparities in psychiatric inpatient outcomes amongst patients with psychotic disorders are known. However, how these two variables intersect to influence features of inpatient care is unclear. We investigated this intersection.

**Methods:**

Using de-identified electronic health data from inpatient services at a large south London mental healthcare provider, we identified a sample of 6767 working-age patients with non-affective psychotic disorders who were admitted between 2016 and 2019. Logistic and negative binomial regressions were used to examine the relationships between ethnicity (and then deprivation) with inpatient-related features (compulsory admission, psychiatric intensive unit admission, length of stay and number of admissions), adjusting for confounders. The sample was stratified by area-level deprivation to understand the intersection of ethnicity, deprivation and these inpatient-related features.

**Results:**

Patients from all areas except the least deprived were at greater risk of compulsory admission, admission to psychiatric intensive care units and more frequent admissions compared with patients from the least deprived areas. All minoritised ethnic patients were more likely to be compulsorily admitted compared with White British patients. Living in the least deprived areas appeared to offer protection against compulsory admission for some ethnic minority groups, but not Black British or Asian patients.

**Conclusions:**

This study showed how psychiatric inpatient-related features for patients with non-affective psychotic disorders were explained not only by the separate effects of area-level deprivation and ethnicity but also by the unique intersections of these two factors. Our findings have implications for policy and interventions aimed at reducing the drivers of inpatient admissions by addressing social stressors in deprived areas and among ethnic minority patients.

**Supplementary Information:**

The online version contains supplementary material available at 10.1007/s00127-025-02908-1.

## Introduction

Non-affective psychotic disorders refer to mental health conditions, including schizophrenia and other related diagnoses, which significantly impact a person’s ability to engage in functional and occupational activities [1]. Globally, the pooled incidence of all psychotic disorders is 26·6 per 100,000 persons [2]. However, the burden of these diagnoses extends beyond the individual to their families and wider society [1, 3, 4].

Psychiatric inpatient admissions have frequently been used as a treatment approach for non-affective psychotic disorders [5]. Admission can be voluntary or involuntary, with the Mental Health Act [6] used to admit patients in the UK compulsorily. Various drivers of compulsory admission and the need for repeated or longer admissions in patients with psychosis have been identified such as a patients lack of insight, disengagement with community care [7, 8], and increased psychotic symptom severity such as delusions [9]. Individuals living in deprived communities experience an ecological concentration of poverty, unemployment, economic disinvestment, and social disorganisation [10], which can place residents at greater risk of mental health difficulties or worsening of illness [11]. Black African, Black Caribbean and other ethnic minority groups in the Global North are at an elevated risk of psychotic disorder and adverse pathways to care [12]. This is partially linked to the social and psychological factors such as discrimination, marginalization and racism they may face [13–15], which limit engagement in community care due to negative experiences or stigma [16].

Ethnic differences in compulsory admission have widely been reported, with Black African and Black Caribbean patients with psychotic disorders more likely to be compulsorily admitted [17–20]. Furthermore, patients from Black ethnic groups are more likely to have a longer length of hospital stay (LOS) [21], be re-admitted [22] and be admitted to psychiatric intensive care units (PICU) compared with White ethnic patients [23]. There is less consensus about the nature of the relationship between deprivation and inpatient-related features. Some studies show that compared with those in less deprived areas, patients with psychotic disorders living in deprived areas experience higher rates of hospital admissions [5, 24] and longer lengths of stay (LOS) [25, 26], and others showed no difference [27] or even that patients from more deprived areas can experience shorter LOS [28].

Developing a more nuanced understanding of which individuals with non-affective psychotic disorders have greater vulnerability to these inpatient-related features is crucial to ensuring resources used to reduce this vulnerability and are targeted appropriately and effectively. Research has typically focused on single sociodemographic factors such as ethnicity, area-level deprivation or gender and inpatient-related features, however unpicking these health inequalities is a complex task characterised by the interplay of the different elements of our identity [29]. Intersectionality acknowledges everyone’s unique experience of discrimination and oppression, but research incorporating this in its design and analysis is limited, likely due to the complex nature of this relationship and the lack of large data sets to address these questions [30]. It is essential to consider how ethnicity and deprivation intersect concerning inpatient-related features, given ethnic minority groups are overrepresented in deprived areas [31] and inpatient settings [17].

This study aims to build on Chow et al. [32], who completed a stratified analysis concerning ethnicity and likelihood of admission in low versus high-poverty areas. Dichotomising deprivation may make data analysis easier but arguably oversimplifies its complexity [33]. This study aimed to operationalise area-level deprivation into quintiles according to the English Indices of Deprivation [34] to give a more detailed description of the relationship between ethnicity, area-level deprivation, and features of inpatient care in individuals with psychotic disorders. We addressed the following research questions:

1. What is the relationship between ethnicity and features of inpatient care in working-age adults with non-affective psychotic disorders?
2. What is the relationship between area-level deprivation and features of inpatient care in working-age adults with non-affective psychotic disorders?
3. How do ethnicity and area-level deprivation impact features of inpatient care among working-age adults with non-affective psychotic disorders?

## Methods

### Study design, setting and data source

We employed a cross-sectional design, using data from the fully de-identified electronic health records of South London and Maudsley (SLaM) NHS Foundation Trust. SLaM is a large mental health provider, serving a catchment of around 1.3 m residents and a caseload of around 45,000 people in contact with services at any time, including inpatient care across 52 inpatient wards [35]. SLaM’s services are provided to four boroughs of south London (Croydon, Lambeth, Lewisham, and Southwark) with a high proportion of residents from ethnic minority backgrounds and relatively high levels of deprivation compared to England overall [36], although a high within-catchment heterogeneity has been observed as shown in Supplementary Table S1. Clinical records were accessed via the Clinical Record Interactive Search (CRIS) system which was set up to allow researchers access to de-identified data within a robust data security and governance framework [37]. Source data for CRIS is drawn from both structured fields (e.g. dates, diagnosis and demographics) and unstructured free-text fields (e.g. case notes and correspondence), with an extensive programme of natural language processing over the last 10 + years to derive relevant meta-data from the latter [36, 38].

### Ethical approval

CRIS was granted ethical approval for secondary research by the South Central- Oxford C Research Ethics Committee (23/SC/0257), and this study was compliant with all elements of the CRIS Security Model [39].

### Case identification and inclusion criteria

Information from structured fields was used to identify patients who met the following inclusion criteria: (a) aged 18–64 (inclusive) at the start of 2016, (b) had a recorded primary or secondary diagnosis of a non-affective psychotic disorder (ICD-10: F20-29) [40] within the study period (2016–2019), (c) had at least one hospital admission to any adult inpatient service in SLaM during 2016–2019. This duration is in line with previous research using CRIS to investigate inpatient use and sociodemographic factors [27], allowing for the identification of sufficient sample size, and the period was chosen as the most recent timeframe avoiding the COVID-19 pandemic, during which inpatient care was atypical [41]. All admissions were considered if someone had multiple admissions over the study period. However, their address from the start of the study period was used.

### Data extraction and measures

#### Sociodemographic data

Sociodemographic data, i.e., age, sex and ethnicity, were extracted from CRIS structured fields. Data extraction was guided by an adapted Medical Research Council Sociodemographic schedule (MRC-SDS) [42].

#### Deprivation

Deprivation was extracted from CRIS structured fields showing participants’ first recorded postcode in the study period, meaning if someone moved during the study period, only their first address would be used. Individual patient residential postcodes within CRIS are linked with area-level deprivation data, as shown by the 2019 English Indices of Deprivation (IMD) [34]. The IMD was calculated for England’s Lower-layer Super Output Area (LSOA), small areas containing between 1,000 and 3,000 residents. Data used for IMD is sourced from administrative data such as benefit records from the Department of Work and Pensions and census data [34]. This study utilised IMD decile scores as a measure of deprivation, collapsed into national quintiles from one (most deprived) to five (least deprived) in line with Reichert and Jacobs [43]. Information on individual-level deprivation (such as employment and participants’ living arrangements) was initially sought from CRIS. However, data was poorly recorded in both the structured and unstructured fields; therefore, personal levels of deprivation were not included this study.

#### Ethnicity

Ethnicity was self-ascribed by patients and recorded in structured fields. Where this was missing (*n* = 343), the researchers manually ascribed ethnicity through the unstructured fields. This was done using the CRIS ‘Front End’ interface (a web-based searchable interface) to retrieve data manually from each patient record [36]. Search terms included “Black”, “White”, “Mixed” and “Asian” to highlight where clinicians documented patients’ ethnicity in case notes. Ten per cent of cases where ethnicity was assigned from free text searches were checked independently by SO, with an agreement rate of 91.43% (K = 0.90) between the raters. In the CRIS database, ethnicity is coded according to the UK census ethnic classifications. For analysis and due to small numbers in some ethnic categories, we collapsed ethnicity into seven larger ethnic groups: White British, White non-British (White Irish, White Gypsy, White Other), Black Caribbean, Black African, Black British, Asian (Indian, Pakistani, Bangladeshi), Mixed (all mixed ethnic groups) and Other (Arab, Chinese, any Other Ethnic group). This process considered guidelines by Ross [44] and followed methods used by Oduola et al. [12], determining the number and composition of ethnic groups based on sample size and descriptive statistics.

#### Inpatient care features and outcome data

Longer length of stay (LOS) [45], compulsory admission [9, 19], PICU admission [46], and number of admissions use of seclusion [47] were chosen as features of inpatient care and the main outcomes in this study. We collected data on the use of seclusion and admission to Forensic Wards and reported these in Table 1: sample characteristics, below. However, we removed these two variables from subsequent analyses due to the small number of patients who experienced these inpatient features.

**Table 1:** Sample characteristics, stratified by area-level deprivation (national IMD quintile)

*N*(%)	1 Most deprived *n* = 1,565	2 *n* = 2,796	3 *n* = 1,204	4 *n* = 393	5 Least deprived *n* = 196	Missing IMD *n* = 612
**Ethnicity** ^**1**^						
White British	502 (32.24)	868(31.27)	512(43.17)	231(59.84)	137(71.35)	
White non-British	146(9.38)	311(11.20)	113(9.53)	37(9.59)	8(4.17*)*	
Mixed	55(3.53)	134(4.843)	47(3.93)	17(4.40)	5(2.60)	
Asian	114(7.32)	184(6.63)	80(6.75)	28(7.25)	18(9.38)	
Black African	150 (9.63)	260(9.37)	95(8.01)	14(3.63)	2(1.04)	
Black Caribbean	232 (14.90)	412(14.84)	120(10.12)	24(6.22)	8(4.17)	
Black British	304(19.52)	522(18.80)	185(15.60)	32(8.29)	11(5.73)	
Other	54(3.47)	85(3.06)	34(2.87)	3(0.78)	3(1.56)	
**Sex** ^**2**^						
Female	718 (45.88)	1,241 (44.40)	580 (48.21)	207 (52.67)	110 (56.12)	
Male	847 (54.12)	1,554 (55.60)	623 (51.79)	186 (47.33)	86 (43.88)	
**Age M(SD)**	37 (12.08)	38 (12.04)	37 (12.26)	36 (12.38)	35 (12.61)	
**Primary Diagnosis**						
Substance-induced psychosis	2 (0.13)	10 (0.36)	4 (0.33)	1 (0.25)	8 (4.08)	
Schizophrenia	398 (25.43)	677 (24.21)	266 (22.09)	49 (12.47)	1 (0.51)	131(21.41)
Delusional disorder	26 (1.66)	43 (1.54)	14 (1.16)	4 (1.02)	6 (3.06)	7(1.14)
Acute psychosis	52 (3.32)	138 (4.94)	50 (4.15)	14 (3.56)	6 (3.06)	31(5.07)
Schizo-affective	148 (9.46)	236 (8.44)	88 (7.31)	21 (5.34)	8 (4.08)	34(5.56)
Unspecified psychosis	204 (13.04)	339 (12.12)	140 (11.63)	36 (9.16)	11 (5.61)	82(13.40)
other	15 (0.96)	18 (0.64)	11 (0.91)	6 (1.53)	2 (1.02)	6(0.98)
Not stated	720 (46.01)	1,335 (47.75)	631 (52.41)	262 (66.67)	160 (81.63)	321(52.45)
**Admission to PICU**						
No	1,361(86.96)	2,403(85.94)	1,065(88.460)	370(94.15)	191(97.45)	
Yes	204(13.04)	393(14.06)	139(11.54)	23(5.85)	5(2.55)	
**Admission to Forensic ward**						
No	1,529(97.70)	2,718(97.21)	1,171(97.26)	387 (98.47)	193(98.47)	
Yes	36(2.30)	78(2.79)	33(2.74)	6 (1.530)	3(1.53)	
**Compulsory admission**						
No	446(28.50)	797(28.51)	425(35.30)	178(45.29)	168(27.45)	
Yes	1,119(71.50)	1,999(71.49)	779(64.70)	215(54.71)	444(72.55)	
**Number of sections Mdn (IQR)**	1 (0–3)	1(0–3)	1(0–2)	1(0–2)	0(0–1)	
**Experienced Seclusion**						
No	1,524(97.38)	2,711(96.96)	1,181(98.09)	389(98.98)	194(98.98)	
Yes	41(2.62)	85(3.04)	23(1.91)	4(1.02)	2(1.02)	
**LOS Mdn (IQR)**	38 (14–103)	40 (15–105)	40(13.5-105.5)	48(15–119)	66.5(24-129.5)	
**No. admissions Mdn (IQR)**	2(1–3)	2(1–3)	1(1-2.5)	1(1–2)	1(1–2)	

Missing records: ^1^ 69 participants, ^2^ 3 participants, IMD 612 participants

The outcome data were extracted from CRIS-structured fields. Length of stay (LOS) was extracted as the cumulative number of days from the date of admission to the date of discharge, cumulative for admissions in the period for each patient. Compulsory admission was coded as a binary present/absent variable according to the use of any section of the MHA on or during admission (including for assessment and treatment). PICU and seclusion were likewise extracted as binary variables according to the use of either of those facilities/interventions. The number of admissions was a count variable, totalled over the study period to give a single value and was chosen as a measure of relapse [48]. Admission to the forensic ward was additionally ascertained as a binary variable as patients’ needs differ from those on non-forensic wards [49]. Forensic wards are used to provide care for offenders with mental health needs whilst reducing their risk of re-offending [50].

### Statistical analysis

Data were analysed using STATA version 15.1 [50]. Descriptive statistics and regression analysis were used. The assumption of multi-collinearity was confirmed using VIF (1.03). Negative binomial regression models were used to overcome the over-dispersion of zero (Pearson goodness-of-fit *X*2 = 1418377, *p* < 0.0001) in the count data. Benjamini and Hochberg’s [51] correction for False Discovery Rate was initially applied to control for multiple comparisons; however, as all *p*-values were still significant after this correction, unadjusted p-values are reported. Below, we set out the analyses we performed to address each research question.

*Q1 What is the relationship between area-level deprivation and features of inpatient care in working-age adults with non-affective psychotic disorders?*

We performed crude and multivariable logistic regression analysis for binary outcomes (compulsory admission and PICU), negative binomial regression for count outcomes (LOS and number of admissions), and adjusted for a-priori confounders (age, sex, and ethnicity). Area-level clustering was controlled by adjusting the standard errors using the *cluster (imd)* command in STATA.

*Q2 What is the relationship between ethnicity and features of inpatient care in working-age adults with non-affective psychotic disorders?*

We built the same regression models as in Q1 above, but we fitted ethnicity as the independent variable.

*Q3 How do ethnicity and area-level deprivation impact on features of inpatient care among working-age adults with non-affective psychotic disorders?*

Data were stratified by area-level deprivation. Within each stratum (deprivation quintile), we assessed associations between ethnicity and compulsory admissions by fitting multivariable logistic regression models, whilst controlling for confounders (age and sex). To examine the association between ethnicity, LOS and number of admissions within each stratum, we employed multivariable negative binomial regression, adjusting for confounders (age and sex). In all regression models, we used the White British ethnic group as the reference group. Given only 12.43% of the sample had experienced a PICU admission (see Supplementary Material 1, Table S2 for whole sample characteristics and Table S3 for sample characteristics stratified by ethnicity), we omitted PICU admission as an outcome variable from the stratified analysis to reduce the risk of type I errors.

Aside from the descriptive statistics reported in Table 1, S2 and S3, all other analyses were conducted with complete data.

**Sensitivity analyses.**

We performed sensitivity analyses (see Supplementary Material 2) by restricting the sample to the patients who had a diagnosis of psychotic disorders recorded (*n* = 3,030). We addressed research questions 1 and 2.

## Results

### Sample characteristics

In total, 6767 eligible patients were identified; of these, 6,095 had complete data. Table 1 shows the sample demographic and clinical characteristics stratified by IMD quintile. In summary, the mean age was similar across IMD quintiles, however older patients [Mean 38 (SD = 12.04) years] resided in the second most deprived area and younger [Mean 35 (SD = 12.61) years] patients were mostly represented in the least deprived areas. Across all IMD quintiles, the largest ethnic group was White British, and they made up the highest proportion of the least deprived quintile (*n* = 137 (71.35%)). Black Caribbean and Black British patients mostly resided in the two most deprived areas. Men were mostly represented in the three most deprived quintiles, whereas most patients were female in the two least deprived areas. Across all deprivation areas, a diagnosis of schizophrenia was common.

### Association between area-level deprivation and features of inpatient care

Focusing on deprivation, first, we estimated the unadjusted and adjusted odds ratios for compulsory admission and then admission to a PICU ward (Table 2). Second, we estimated the unadjusted and adjusted incidence rate ratios for the LOS and number of admissions (Table 2). The least deprived area (Quintile 5) was the comparator group.

**Table 2:** Unadjusted and adjusted odds ratios of associations between area-level deprivation and compulsory admission and admission to PICU, controlling for area-level clustering

Compulsory admission			PICU	
Deprivation quintile	Unadjusted OR Model 1	Adjusted OR Model 2	Unadjusted OR Model 1	Adjusted OR Model 2
4	1.51(1.51–1.51) ***	1.32(1.29–1.35)***	2.37(2.37–2.37)***	2.17(2.13–2.21)***
3	2.30(2.30–2.30) ***	1.72(1.67–1.77) ***	4.99(4.99–4.99) ***	3.73(3.63–3.84)***
2	3.14(3.14–3.14) ***	2.00(1.92–2.08) ***	6.25(6.25–6.25) ***	4.20(4.04–4.37) ***
1 Most deprived	3.14(3.14–3.14) ***	2.00(1.94–2.06) ***	5.73(5.73–5.73) ***	3.78(3.66–3.92) ***

*<0.05, **<0.01, ***<0.001 Comparison group = quintile 5 (least deprived) Model 2: adjusted for age, gender, ethnicity

### Compulsory admission

We found strong evidence that patients living in deprivation quintiles 1 to 4 were between 1.3 and 1.9 times more likely to be compulsorily admitted compared with those living in the least deprived quintile (quintile 5).

### PICU admission

In both the unadjusted and adjusted models, there was strong evidence that patients living in quintiles one, two and three were 2.2–4.2 times more likely to be admitted to a PICU than their quintile 5 counterparts (see Table 2).

### LOS

Compared with those in the least deprived areas (quintile 5), patients living in other deprivation areas (i.e. quintile 1 to 4) had shorter LOS, as shown in Table 3.

**Table 3:** Unadjusted and adjusted incidence rate ratios of associations between area-level deprivation and length of stay and number of admissions, controlling for area clustering

	LOS		Number of admissions	
Deprivation quintile	Unadjusted IRR Model 1	Adjusted IRR Model 2	Unadjusted IRR Model 1	Adjusted IRR Model 2
4	0.76(0.76-0.7.6) ***	0.75(0.61-0.67)***	1.60(1.03–1.43) ***	1.45(1.43–1.48) ***
3	0.71(0.71-0.71) ****	0.62(0.59-0.65) ***	1.60(1.60–1.60) ***	1.46(1.43–1.493) ***
2	0.75(0.75-0.75 ***	0.62(0.59-0.63 ***	1.50(1.50–1.50) ***	1.41(1.39–1.43) ***
1Most deprived	0.76(0.76-0.76) ***	0.64(0.65-0.6.75) ***	1.21(1.21–1.21) ***	1.19(1.18–1.20) ***

*<0.05, **<0.01, ***<0.001 Comparison group = quintile 5. Model 2: adjusted for age, gender, ethnicity

### Number of admissions

There was strong evidence that patients living in quintiles 1 to 4 had a higher number of admissions compared with those in quintile 5, even after controlling for confounders, as shown in Table 3.

### Sensitivity analysis

In our sensitivity analysis focusing only on patients with a record of primary diagnosis of psychosis, we observed consistent trends with our main analysis in terms of deprivation and admission to a PICU ward, number of admissions and LOS. However, for deprivation and compulsory admission, the odds of compulsory admission among patients living in deprived areas had diminished relative to those living in the least deprived areas (See Supplementary Material 2, Tables S5a-b).

### Association between ethnicity and features of inpatient care

First, we estimated the unadjusted and adjusted odds ratios for compulsory admission and then admission to a PICU ward (Table 4). Second, we estimated the incidence rate ratios for the LOS and the number of admissions (Table 4). White British ethnicity was the comparator group.

**Table 4:** Unadjusted and adjusted odds ratios of associations between ethnicity and compulsory admission and admission to PICU

Compulsory admission			Admission to PICU	
Ethnicity	Unadjusted OR Model 1	Adjusted OR Model 2	Unadjusted OR Model 1	Adjusted OR Model 2
White non-British	1.92(1.59–2.32) ***	1.85(1.53–2.34) ***	1.51(1.08–2.01) *	1.32(0.94-1.84)
Mixed	1.90(1.45–2.51) ***	1.90(1.44–2.51) ***	2.96(2.03–4.31) ***	2.48(1.69–3.65) ***
Asian	2.31(1.84–2.91) ***	2.29(1.82–2.89) ***	1.54(1.06–2.24) *	1.36(0.93 − 2.00)
Black African	4.07(3.20–5.43) ***	3.61(2.83–4.60) ***	3.18(2.36–4.24) ***	3.18(2.37–4.29) ***
Black Caribbean	4.43(3.61–5.43) * **	4.18(3.40–5.15) ***	3.94(3.01–5.03) ***	3.53(2.75–4.5) ***
Black British	3.99(3.34–4.77) ***	3.78(3.15–4.53) ***	4.49(3.58–5.63) ***	3.68(2.92–4.64) ***
Other	1.61(1.17–2.21) **	1.54(1.17–2.12) **	0.93(0.48-1.79)	0.74(0.38-1.44)

*<0.05, **<0.01, ***<0.001. Comparison group = White British Model 2: adjusted for age, gender, deprivation IMD

### Compulsory admission

We found strong evidence that all minoritised ethnic groups were between 1.5 and 4.1 times more likely to be admitted compulsorily compared with White British patients independent of confounders, as shown in Table 4.

### PICU admission

In the unadjusted odds ratio, all ethnic minority groups except ‘other’ ethnic group patients were more likely to be admitted to PICU, compared with White British patients. However, after controlling for confounders, the strength of this association only remained for Black African, Black Caribbean, Black British and Mixed ethnic group patients, as shown in Table 4.

### LOS

In both the unadjusted and adjusted incidence rate ratios, Black African, Black Caribbean and Black British patients were more likely to have a longer LOS in comparison to White British patients. White non-British and patients from other ethnic groups had a shorter LOS (see Table 5).

**Table 5:** Unadjusted and adjusted incidence rate ratios of associations between ethnicity and length of stay and number of admissions

	LOS		Number of admissions	
Ethnicity	Unadjusted IRR Model 1	Adjusted IRR Model 2	Unadjusted IRR Model 1	Adjusted IRR Model 2
White non-British	0.813(0.73-0.91)***	0.86(0.77–96)**	1.01(9.30–1.84)	0.97(0.90-1.05)
Mixed	1.11(0.95 − 1.30)	0.1.15(0.98-1.35)	1.223(1.11–1.37) ***	1.18(1.07–1.32) *
Asian	1.00(0.88-1.14)	1.03(0.91-1.17)	1.0(0.92-1.09)	0.98(0.90-1.07)
Black African	1.61(1.43–1.81) ***	1.59(1.41–1.78) ***	1.30(1.21–1.41) ***	1.27(1.18–1.37) ***
Black Caribbean	1.27(1.12–1.39) ***	1.31(1.88–1.45) ***	1.35(1.27–1.45) ***	1.30(1.22–1.39) ***
Black British	1.329(1.21–1.45) ***	1.38(1.26–1.51) ***	1.44(1.36–1.53) ***	1.38(1.30–1.47) ***
Other	0.579.47-0.69) ***	0.62(0.52-0.75) ***	0.89(0.78-1.02)	0.85(0.74-0.98) *

*<0.05, **<0.01, ***<0.001. Comparison group = White British. Model 2: adjusted for age, gender, deprivation IMD

### Number of admissions

Mixed ethnicity, Black African, Black Caribbean and Black British patients were more likely to experience multiple admissions. However, we found evidence that patients from ‘Other’ ethnic groups were admitted less frequently (see Table 5).

### Sensitivity analysis

Our sensitivity analyses showed that the odds of compulsory admission by ethnicity was strong for only Black Caribbean and Black British patients. In terms of number of admissions and ethnicity, findings from the sensitivity and main analyses were consistent. However, we observed that Black Caribbean and Black British were no longer at risk of a longer LOS (See Supplementary Material 2, Tables S5c-d).

### Intersection between ethnicity, deprivation, and features of inpatient care

**Fig. 1:**
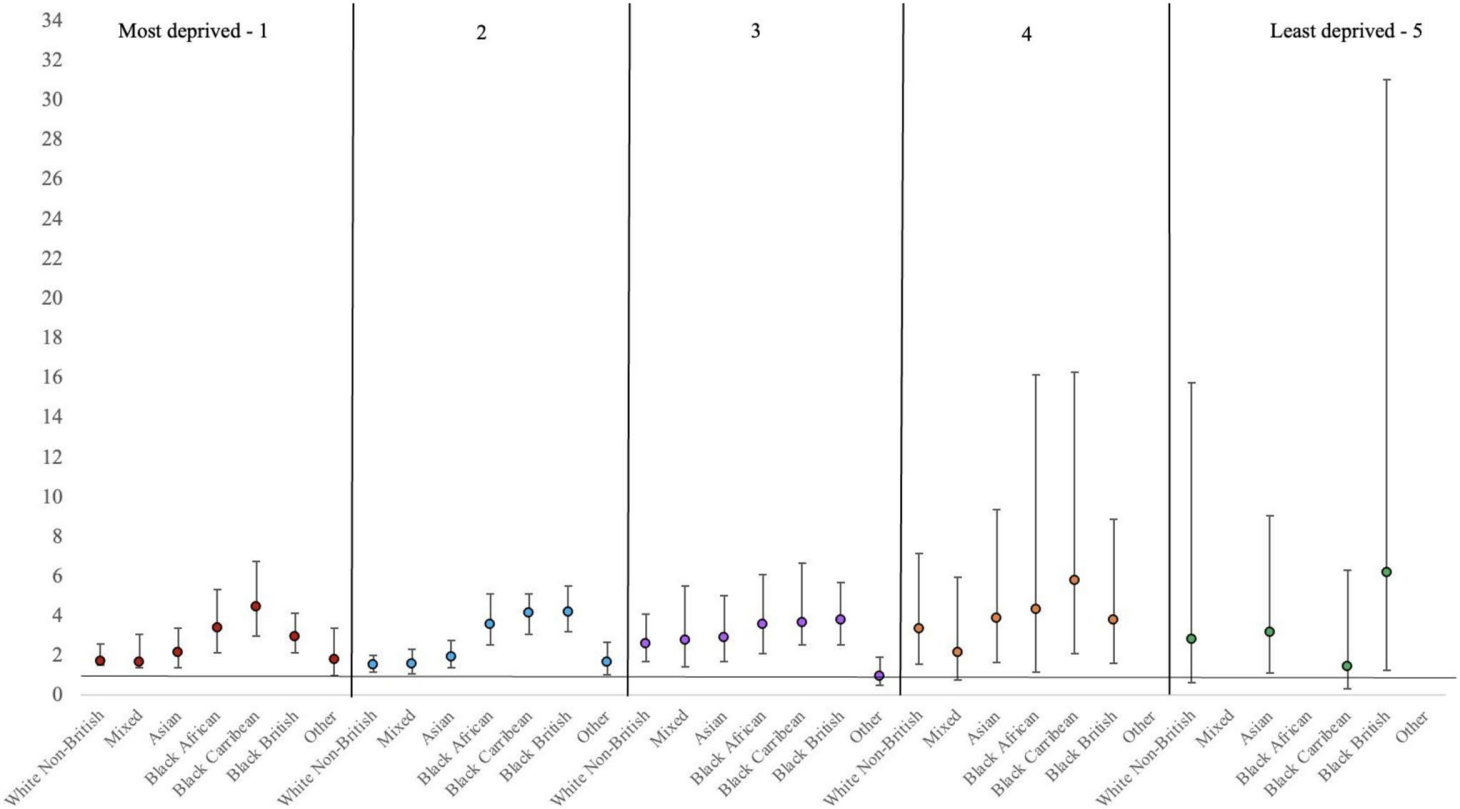
Adjusted Odds ratio (OR) and their corresponding 95% confidence intervals (CI) from logistic regressions assessing the association between ethnicity and compulsory admission, stratified by area-deprivation

**Fig. 2:**
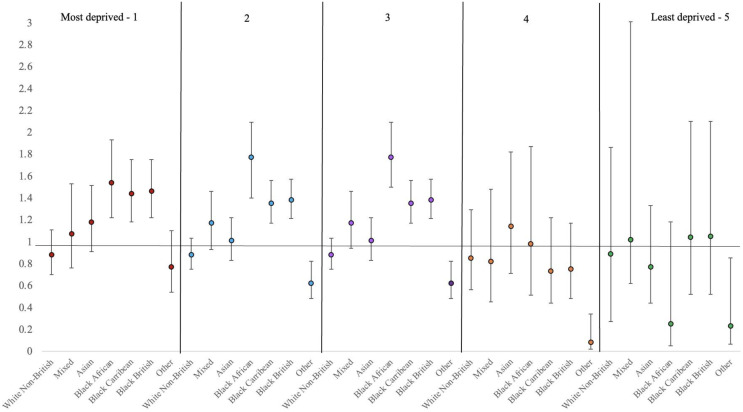
Adjusted incidence rate ratios (IRR) and their corresponding 95% confidence intervals (CI) from negative binomial regressions assessing the association between ethnicity and LOS, stratified by area deprivation

**Fig. 3:**
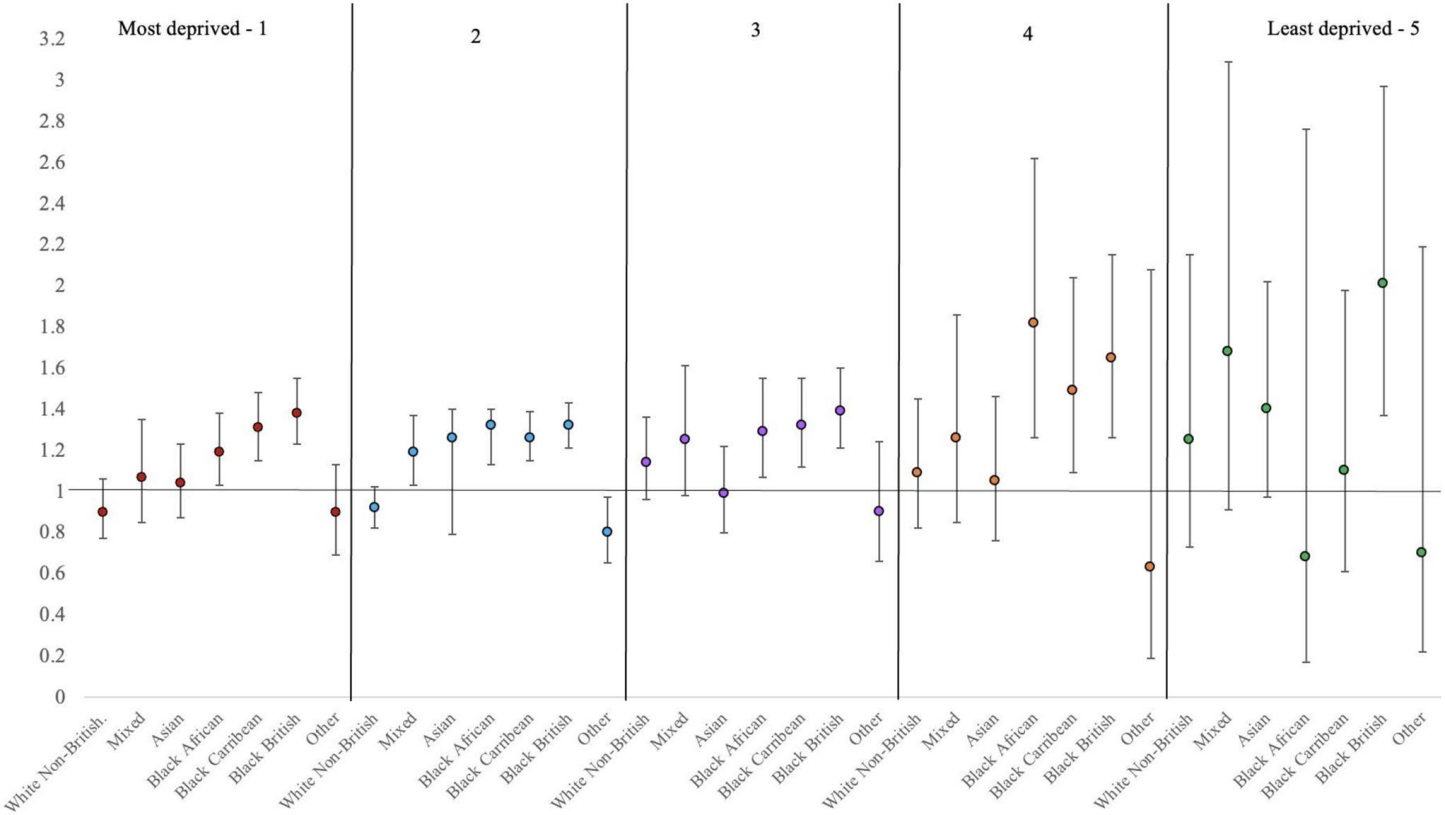
Adjusted incidence rate ratios (IRR) and their corresponding 95% confidence intervals (CI) from negative binomial regressions assessing the association between ethnicity and number of admissions, stratified by area deprivation

Supplementary Table S4 shows the effect sizes of associations between ethnicity and inpatient outcomes stratified by deprivation quintiles in table form, and in Figs. 1, 2 and 3 below, we present the graphical illustrations.

### Compulsory admission

Across the first four quintiles and compared with White British patients, White Non-British, Asian, and Black (African, Caribbean and British) patients were (between 1.7 and 5.7 times) more likely to be admitted compulsorily as shown in Fig. 1. This was also true for mixed-ethnicity patients living in quintiles 2 and 3. In quintile five (least deprived area), only Black British and Asian patients were 3 to 6 more likely to be compulsory admitted.

### LOS

In the three most deprived areas (quintiles 1 to 3), LOS was between 1.3 and 1.7 times higher in Black (African, Caribbean, and British) compared with White British patients living in the same quintiles. In quintiles 2, 3, 4 and 5, patients of ‘Other’ ethnic background had shorter LOS compared to their White British counterparts.

### Number of admissions

Black (African, Caribbean, and British) ethnic groups in quintiles 1 to 4 were between 1.2 and 1.8 times more likely to experience multiple admissions compared to White British patients living in the same quintiles. In quintile 2, patients of ‘Other’ ethnic background were admitted less frequently compared to their White British counterparts. In quintile five, only Black British patients were 2 times more likely to be admitted frequently.

## Discussion

### Main findings

This study aimed to understand the relationship between ethnicity, deprivation, and inpatient-related features in working-age adults with non-affective psychotic disorders. We found that patients living in the four most deprived areas (quintiles 1, 2, 3 and 4) were more likely to be compulsorily admitted and experienced more frequent hospital admissions but shorter LOS than those living in quintile 5 (least deprived areas). All minoritised ethnic patients were more likely to be compulsorily admitted than the White British group. Patients from Black ethnic backgrounds (i.e. Black African, Black Caribbean, and Black British) were also more likely to be admitted to a PICU, experienced longer LOS, and had a higher number of admissions.

All minoritised ethnic groups in the three most deprived areas (quintiles 1, 2 and 3) were more likely to experience compulsory admission. Conversely, living in the least deprived areas (quintile 5) reduces the risk of compulsory admission for some ethnic minority groups, but not Asian or Black British patients. However, the small sample size and wide confidence intervals within this quintile make this a tentative conclusion. Increased risk for readmissions were present for Black British, Caribbean and African patients living in quintiles 1, 2, 3 and 4 and for Mixed-ethnicity patients in quintile 2. Except for Black British patients, there was no evidence that other ethnic minority patients experienced more frequent hospital admissions than their White British counterparts in the least deprived (quintile 5) areas. Additionally, only in the three most deprived areas (quintiles 1,2 and 3) were Black British, Caribbean, and African patients more likely to have a longer LOS.

### Explaining the findings

When considering deprivation alone, patients living in the least deprived areas were less likely to experience all outcomes, expect a longer length of stay, echoing Hodgson et al. [28], whereby patients living in less deprived areas were more likely to experience a longer LOS. This finding was surprising as higher symptom severity has been associated with longer LOS [52] and the social causation hypothesis would assume residents of deprived areas experience greater social stressors contributing to severe symptomatology requiring longer inpatient treatment [45]. Research in the US has shown that patients with psychotic disorders in low-income areas have shorter hospital admissions than those in higher-income areas [52]. In the UK, NHS patients from the three most deprived quintiles have been reported to be more likely to self-discharge against medical advice, leading to shorter admissions [53], however this is yet to be investigated specifically in patients with psychotic disorders. Moreover, we found that patients from more deprived areas were more likely to be readmitted, despite their admissions being shorter. This could highlight that a longer LOS for patients from less deprived areas could be protective against poor post-discharge outcomes which require readmission.

We found all minoritised ethnic groups were more likely to be compulsorily admitted compared with White British patients. Previous studies have consistently found an elevated Black ethnic patients with psychotic disorders are more vulnerable to compulsory admission [17, 18, 20, 54], however, this finding is less consistent for other ethnic minority groups. Indeed, Freitas [17] also found an elevated risk of Asian Bangladeshi patients, whereas Mohan [18] did not. Individuals who seek help during subclinical stages of psychosis are less likely to be compulsorily admitted for treatment compared to those who do not seek help [55]. Research has shown that help-seeking behaviour in Black ethnic groups can be influenced by fear of punitive treatments [56], stigma [16] and cultural and spiritual interpretations of illness [57]. When Black ethnic patients with psychosis do seek help, they are less likely to be offered psychological interventions [17, 58], which have been associated with reduced rates of compulsory admission [15]. Previous reviews have found evidence to support that racial bias in the perception of dangerousness can influence how clinicians manage patients, often turning to more restrictive interventions like compulsory admission [59]. Therefore, disparities in compulsory admission could be seen to reflect disparities in access to or quality of community care.

A previous stratified analysis by Chow et al. [32] found ethnic minority patients living in low-poverty areas of New York only were more likely to be admitted compared to White patients. Strikingly, we found Black British and Asian patients living in the least deprived areas (quintile five) were also more likely to be compulsorily admitted, though there is a need to interpret these findings cautiously. In our sample, ethnic minority groups were less concentrated in quintile five, reflecting previous research [31]. Own-group ethnic density can be protective against potential drivers of compulsory admission for some ethnic minority groups [60], these areas experience less racism [64]. Racism can shape individuals’ schemas about themselves, the world, and others [61]. Individuals may experience their physical and sociocultural environment as hostile, fuelling more severe psychotic symptoms like persecutory paranoid delusions [62]. Black ethnic people can also experience racism when trying to access healthcare, finding this experience discriminatory and stigmatising, perhaps making them reluctant to engage in outpatient care [16] or accept voluntary admission when experiencing psychotic symptomatology [63], possibly placing them at greater risk of compulsory admission.

The varying levels of racism different ethnic minority groups report could partially explain why patients from other ethnic minority groups living in the least deprived area were at no greater risk of compulsory admission. Individuals from Black ethnic groups have more frequently reported experiences of racism compared with most White non-British individuals [64]. This links to a specific type of discrimination called Colourism, whereby those with lighter skin are privileged [65]. Colourism has been found to contribute to individuals with lighter skin having greater opportunities in areas like employment [66] and in the rental market [67]. This suggests that White non-British patients may face fewer barriers in accessing the beneficial characteristics of least deprived areas compared to Black British patients, which could have a buffering effect against drivers of compulsory admission.

### Methodological considerations

Our study has several methodological strengths, including access to a large and diverse sample of the population of Southeast London through the CRIS data source. This allowed us to disaggregate ethnicity according to the census categories and to stratify area-level deprivation into more groups than in previous studies [32]. However, our sample can be considered London-centric, with a patient sample likely to be more diverse than other UK areas. Access to source text permitted improved ascertainment of ethnicity-related data from the free-text fields for an additional 343 patients who would have been excluded otherwise.

Despite the large sample size, fewer ethnic minority patients lived in the least deprived quintile. This might explain why certain ethnic minority groups had no compulsory admissions or admissions to a PICU in quintile five and why there were larger confidence intervals in groups that did, limiting the strength of conclusions in this quintile. The data in CRIS is recorded by clinicians for clinical, and not necessarily for research purposes. Therefore, the availability and accuracy of information depends on the quality of their information-gathering documentation. This meant there was insufficient data on person-level deprivation factors like employment status to supplement the area-level deprivation measure we used. Given the cross-sectional nature of this study, changes in residential addresses were not accounted for, limiting our ability to infer causality. Future studies will benefit from taking a longitudinal approach to provide temporal insight on this subject.

This study used operationalised area-level deprivation using the IMD [34]. Small-area measures can be subject to ecological fallacy as one might assume that if an area is deprived, all residents living there will be deprived. However, research has found differences between an individual’s actual financial resources and their concern about their relative deprivation [68]. Residents who identify as less deprived may feel they have better access to resources, allowing them to mitigate some of the disadvantage characteristics of their community, like having resources to travel to areas with green space. Future research should consider additional individual-level co-founders such as education level or employment status, possibly giving a better understanding of the intricacies of the data.

### Implications of findings

Our findings emphasise the impact of not addressing health inequalities in the drivers of inpatient admissions, leaving people from deprived areas and ethnic minority groups with psychotic disorders potentially more vulnerable to involuntary and frequent hospitalisation. In the UK, the introduction of Integrated Care Systems holds promise for greater collaboration between health services, local authorities, and voluntary third-sector partners [69]. This study has implications for how these systems could target their attempts to reduce these inequalities. One possible approach is for policymakers and service providers to shift the focus away from individual-focused to evidence-based community-level interventions that address potential drivers of hospital admission. Evidence suggests that community-level interventions, such as community support for parents [70, 71] and improved access to community exercise facilities [72, 73], benefit common mental health outcomes in deprived areas and among ethnic minority groups. However, more evidence for these interventions is needed for individuals with psychotic disorders from these communities.

We found that in the least deprived areas, some ethnic minority residents are still at greater risk of compulsory admission. This supports recommendations by the Fair Society, Healthy Lives Review [74], emphasising that focusing solely on the most deprived areas will not sufficiently reduce health inequalities. Instead, resources should be targeted with a scale and intensity proportionate to the level of disadvantage residents face [74]. For example, resources aimed at enhancing community cohesion and addressing experiences of racism may be most effectively target drivers for admission across the spectrum of deprivation. Additional resources aimed at addressing the drivers of admission linked to deprivation may be best targeted at more deprived areas. Such approach has the potential to reduce the increased risk of compulsory admission for ethnic minority patients with psychotic disorders across the deprivation spectrum.

## Conclusions

This study investigated how features of psychiatric inpatient care for patients with psychotic disorders were explained not only by the separate effects of area-level deprivation and ethnicity but also by the unique intersections of these two factors. Our findings show inpatient treatment inequalities are ubiquitous by ethnicity and social characteristics. Evidence-based community-level interventions to tackle health inequalities in the drivers for admission should be a priority for future research, policymakers and service providers.

## Electronic supplementary material

Below is the link to the electronic supplementary material.

Supplementary Material 1

Supplementary Material 2

## Data Availability

No new datasets were generated or analysed during the current study.
