# Supplementary material for: Understanding the intersections between ethnicity, area-level deprivation, and inpatient-related features amongst patients with psychotic disorders: a mental health electronic records analysis
Source: Soc Psychiatry Psychiatr Epidemiol. 2025 May 5;60(8):1957–69. doi: 10.1007/s00127-025-02908-1 (PMC12325388; doi:10.1007/s00127-025-02908-1)
Supplement: Supplementary file 1 — Supplementary Material 1 [file 127_2025_2908_MOESM1_ESM.docx]

Title: Understanding the intersections between ethnicity, area-level deprivation and inpatient-related features amongst patients with psychotic disorders: a mental health electronic records analysis

**Supplementary material 1**

**Table S1:** Comparing ethnicity and deprivation in SLaM areas with England (information taken from Census data 2011).

|  | Lambeth | Lewisham | Croydon | Southwark | England |
| --- | --- | --- | --- | --- | --- |
| **Ethnicity** |  |  |  |  |  |
| White | 55% | 51.5% | 48.4% | 51.4% | 81.0% |
| Mixed | 8.1% | 8.1% | 7.6% | 7.2% | 3.0% |
| Asian | 7.3% | 9.0% | 17.5% | 9.9% | 9.6% |
| Black | 24% | 26.8% | 22.6% | 25.1% | 4.2% |
| Other | 5.7% | 4.7% | 3.9% | 6.3% | 2.2% |
| **Deprivation** |  |  |  |  |  |
| Unemployed | 4.6% | 4.9% | 4.1% | 4.6% | 2.9% |
| Social renting | 33.6% | 29.2% | 17.9% | 39.7% | 17.1% |
| No qualifications | 13.1% | 14.6% | 16.1% | 13.9% | 18.1% |

**Supplementary material 2**

**Table S2**: Sample characteristics

| **Ethnicity** | **Number (%)** |
| --- | --- |
| White British | 2,416 (36.07) |
| White non-British | 775 (11.57) |
| Mixed | 278(4.15) |
| Asian | 468 (6.99) |
| Black African | 566 (8.30) |
| Black Caribbean | 863(12.88) |
| Black other | 1,124(16.78) |
| Other | 218 (3.25) |
| **Gender** |  |
| Female | 3,061(45.25) |
| Male | 3,7061(54.75) |
| **Age M(SD)** | 37(12.06) mean (SD) |
| **IMD quintile** |  |
| 1 Most | 1,565 (25.43) |
| 2 | 2,796 (45.43) |
| 3 | 1,204 (19.56) |
| 4 | 393 (6.39) |
| 5 Least | 196 (3.18) |
| **Primary Diagnosis** |  |
| Substance-induced psychosis | 17(0.25) |
| Schizophrenia | 1,529(22.60) |
| Delusional disorder | 95(1.40) |
| Acute psychosis | 291(4.30) |
| Schizo-affective | 535(7.91) |
| Unspecified psychosis | 812(12) |
| other | 58(0.86) |
| Not stated | 3,2429(50.68) |
| **PICU** |  |

| No | 5,925(87.57) |
| --- | --- |
| Yes | 841(12.43) |
| **Forensic** |  |
| No |  |
| Yes |  |
| **Compulsory admission** |  |
| No | 2,123(31.38) |
| Yes | 4,643(68.62) |
|  |  |
| **Seclusion** |  |
| No | 1,524(97.38) |
| Yes | 41(2.62) |
| **Length of stay Mdn(IQR)** | 41(15-109) |
| **Number of admissions Mdn(IQR)** | 1(1-3) |

**Supplementary material 3**

**Table S3**: Sample characteristics stratified by ethnicity.

White British

| *N*(%) African | | | | | | Caribbean | British | *n*=218 |
| --- | --- | --- | --- | --- | --- | --- | --- | --- |
|  | *n*=2,416 | *n*=775 | *n*=278 | *n*=468 | *n*=566 | *n*=863 | *n*=1,124 |  |
| **Deprivation^1^**  1 | 502(22.31) | 146(23.74) | 55(21.32) | 114(26.89) | 150(28.79) | 232(29.15) | 304(28.84) | 54(30.17) |
| 2 | 868(38.58) | 311(50.57) | 134(51.94) | 184(43.40) | 260(49.90) | 412(51.76) | 522(49.53) | 85(47.49) |
| 3 | 512(22.76) | 113(18.37) | 47(18.22) | 80(18.87) | 95(18.23) | 120(15.08) | 185(17.55) | 34(18.99) |
| 4 | 231(10.27) | 37(6.02) | 17(6.59) | 28(6.60) | 14(2.69) | 24(3.02) | 32(3.04) | 3(1.68) |
| 5 | 137(6.09) | 8(1.30) | 5(1.94) | 18(4.25) | 2(0.38) | 8(1.01) | 11(1.04) | 3(1.68) |
| **Gender^2^** |  |  |  |  |  |  |  |  |
| Male | 1,271(52.65) | 432(55.74) | 143(51.44) | 261(55.77) | 323(58.09) | 455(52.72) | 662(58.90) | 120(55.05) |
| Female | 1,143(47.35) | 343(44.26) | 135(48.56) | 207(44.23) | 233(41.91) | 408(47.28) | 462(41.10) | 98(44.95) |
| **Age M(SD)**  **Primary Diagnosis** | 38(12.44) | 36(11.42) | 34(10.87) | 36(11.85) | 43(12.33) | 37(11.23) | 35(11.59) | 34(11.22) |

White Non-BritishMixed

Asian

BlackBlack

Black

Other

| Substance- induced.  psychosis |  | 3(0.39) |  |  | 2(0.36) | 4(0.46) | 2(0.18) | 21(9.63) |
| --- | --- | --- | --- | --- | --- | --- | --- | --- |
|  | 3(0.12) |  | 1(0.36) | 2(0.43) |  |  |  |  |
| Schizophrenia | 334(13.82) | 149(19.23) | 68(24.46) | 90(19.23) | 235(42.27) | 272(31.52) | 359(31.94) | 3(1.38) |
| Delusional  disorder | 32(1.32) | 10(1.29) | 4(1.44) | 6(1.28) | 9(1.62) | 14(1.62) | 16(1.42) | 8(3.67) |
| Acute psychosis | 67(2.77) | 39(5.03) | 7(2.52) | 25(5.34) | 22(3.96) | 51(5.91) | 67(5.96) | 8(3.67) |
| Schizo-affective | 114(4.72) | 45(5.81) | 21(7.55) | 28(5.98) | 64(11.51) | 112(12.98) | 136(12.10) | 14(6.42) |
| Unspecified  psychosis | 162(6.71) | 103(13.29) | 40(14.39) | 65(13.89) | 59(10.61) | 142(16.45) | 197(17.53) | 37(16.97) |
| other | 23(0.95) | 8(1.03) | 2(0.72) | 3(0.64) | 4(0.72) | 5(0.58) | 12(1.07) | 1(0.46) |
| Not stated |  |  |  |  |  | 263(30.48) | 335(29.80) | 134(61.47) |
|  | 1,681(69.58) | 418(53.94) | 135(48.56) | 249(53.21) | 161(28.96) |  |  |  |
| **Admission to**  **PICU** |  |  |  |  |  |  |  |  |
| No | 1,361(86.96) | 2,403(85.94) | 234(84.17) | 428(91.45) | 466(83.81) | 690(79.95) | 869(77.31) | 204(93.58) |
| Yes | 204(13.04) | 393(14.06) | 44(15.83) | 40(8.55) | 90(16.19) | 173(20.05) | 255(22.69) | 14(6.42) |
| **Admission to**  **Forensic** |  |  |  |  |  |  |  |  |

| No | 2,377(98.39) |  | 268(96.40) | 459(98.08) | 520(93.53) | 828(95.94) | 1,062(94.4  8) | 215(98.62) |
| --- | --- | --- | --- | --- | --- | --- | --- | --- |
|  |  | 758(97.81) |  |  |  |  |  |  |
| Yes | 39(1.61) | 17(2.19) | 10(3.60) | 9(1.92) | 36(6.47) | 35(4.06) | 62(5.52) | 3(1.38) |
| **Compulsory admission** |  |  |  |  |  |  |  |  |
| No | 1,113(53.93) | 237(30.58) | 85(30.58) | 128(27.35) | 97(17.45) | 142(16.45) | 200(17.79) | 71(32.57) |
| Yes | 1,303(53.93) | 538(69.42) | 193(69.42) | 340(72.65) | 459(82.55) | 721(83.55) | 924(82.21) | 147(67.43) |
| **Number of sections**  **Mdn(IQR)** | 1 (2) | 1(2) | 1(3) | 1(2) | 2(2) | 2(2) | 2(2) | 1(2) |
| **Experienced**  **Seclusion** |  |  |  |  |  |  |  |  |
| No | 2,401(99.38) | 768(99.10) | 266(95.68) | 460(98.29) | 537(96.58) | 823(95.37) | 1,062(94.4  8) | 213(97.710 |
| Yes | 15(0.62) | 7(0.90) | 12(4.32) | 8(1.71) | 19(3.42) | 40(4.63) | 62(5.52) | 5(2.29) |
| **LOS**  **Mdn(IQR)** | 34.5(91) | 30(63) | 36.5(90) | 38(87) | 64(149) | 55(112) | 54.5(112) | 26.5(46) |
| **No. admissions**  **Mdn(IQR)** | 1(3) | 1(1) | 2(2) | 1(1) | 2(2) | 2(2) | 2(3) | 1(1) |
| Missing records: ^1^ 69 participants, ^2^ 3 participants, IMD 613 participants | | | | |  |  |  |  |

**Supplementary material 4**

**Table S4**: Odds ratios and Incident rate ratios for intersectionality between ethnicity and area-level deprivation.

| IMD quintile |  | LOS |  | Compulsory admission |  | Number of admissions | |
| --- | --- | --- | --- | --- | --- | --- | --- |
|  |  | Unadjusted | Adjusted | Unadjusted | Adjusted | Unadjusted Adjusted | |
| **1Least** | White non- British | .89(.70-1.11) | .88(.70-1.11) | 1.72(1.16-  2.56)** | 1.71(1.15-  2.55)** | .90(.771.06) | .90(.77-1.06) |
|  | mixed | 1.01(.76-1.52) | 1.07(.76-1.53) | 1.66(.91-3.02) | 1.66(.912-3.04) | 1.09(.86-  1.37) | 1.07(.85-  1.35) |
|  | Asian/south  Asian | 1.19(.92-1.54) | 1.18(.91-1.512) | 2.17(1.38-  3.44)** | 2.13(1.35-  3.38)** | 1.93(.87-  1.22) | 1.04(.87-  1.23) |
|  | Black African | 1.57(1.25-  1.97)*** | 1.54(1.22-  1.93)*** | 3.29(2.15-  5.32)*** | 3.38(1.35-  3.38)*** | 1.12(1.01-  1.36)* | 1.19(1.03-  1.23)* |
|  | Black  Caribbean | 1.46(1.20-  1.78)*** | 1.44(1.18-  1.75)*** | 4.48(2.98-  6.74)*** | 3.38(2.14-  5.33)*** | 1.31(1.24-  1.56)*** | 1.31(1.15-  1.48)*** |
|  | Black British | 1.43(1.20-  1.71)*** | 1.46(1.22-  1.75)*** | 2.96(2.12-  4.12_*** | 4.46(2.96-  6.71)*** | 1.39(1.24-  1.56)*** | 1.38(1.23-  1.55)*** |
|  | Other | .74(.52-1.05) | .77(.54-1.10) | 1.76(.96-3.25) | 1.81(.98-3.35) | .90(.71-  1.16) | .89(.6**9-1.13)** |
| **2** | White non-  British | .83(.71-.97)* | .88(.75-1.03) | 1.48(1.13-  1.95)** | 1.53(1.16-  2.001)** | .92(.83-  1.03) | .92(.82-1.02) |
|  | mixed | 1.08(.87-1.36) | 1.42(.98-1.05) | 1.45(.99-2.13) | 1.57(1.062-  2.31)* | 1.21(1.05-  1.39)** | 1.19(.103-  1.37)* |
|  | Asian/south  Asian | .96(.79-1.16) | .99(.74-1.31) | 1.86(1.32-  2.64)*** | 1.93(1.36-  2.74)*** | .92(.80-  1.05) | .90(.79-1.04) |
|  | Black African | 1.88(1.59-  2.26)**** | 1.42(1.08-  1.86))*** | 3.70(2.600-  5.27)*** | 3.57(2.50-  5.09)*** | 1.26(1.3-  1.40)*** | 1.26(1.13-  1.40)*** |
|  | Black  Caribbean | 1.31(1.14-  1.51)*** | 1.31(1.03-  1.67)*** | 3.70(2.60-  5.28)*** | 4.14(3.06-  5.08)*** | 1.27(1.16-  1.39)*** | 1.26(1.15-  1.39)*** |

|  | Black British | 1.37(1.20-  1.56)*** | 1.54(1.25-  1.89)*** | 4.05(3.08-  5.33)*** | 4.18(3.17-  5.51)*** | 1.34(1.23-  1.46)*** | 1.32(1.21-  1.34)*** |
| --- | --- | --- | --- | --- | --- | --- | --- |
|  | Other | .56(.43-  .74)*** | .53(.35-.82)** | 1.58(.98-2.54) | 1.64(1.02-  2.64)* | .81(.67-  .99)* | .80(.65-.97)* |
| **3** | White non-  British | .90(.70-1.15) | .88(.75-1.03) | 2.46(1.57-  3.84)*** | 2.60(1.66-  4.09)*** | 1.16(.97-  1.38) | 1.41(.96-  1.36) |
|  | mixed | 1.64(1.14-  2.37)** | 1.17(.94-1.46) | 2.71(1.37-5.34)  ** | 2.78(1.40-  5.47)** | 1.26(.98-  1.62) | 1.25(.98-  1.61) |
|  | Asian/south  Asian | .99(1.14-2.37) | 1.01(.83-1.22) | 2.78(1.63-  4.76)*** | 2.90(1.70-  4.98)*** | .99(.81-  1.23) | .99(.80-1.22) |
|  | Black African | 1.58(1.21-  2.07)** | 1.77(1.50-2.09)* | 3.71(2.18-  6.32)*** | 2.91(1.70-  4.98)*** | 1.27(1.06-  1.53)** | 1.29(1.07-  1.55)** |
|  | Black  Caribbean | 1.37(1.07-  1.75) * | 1.35(1.17-1.56)* | 3.52(2.20-  5.66)*** | 3.56(2.09-  6.08)*** | 1.33(1.13-  1.57)*** | 1.32(1.12-  1.55)** |
|  | Black British | 1.61(1.31-  1.98)*** | 1.38(1.21-  1.57)*** | 3.59(2.42-  5.34)*** | 3.78(2.53-  5.65)*** | 1.43(1.24-  1.64)*** | 1.39(1.21-  1.60)*** |
|  | Other | .57(.37-  .87)0.010** | .62(.48-.82)** | .923(.46-1.86) | .94(.469-1.89) | .91(.67-  1.26) | .90(.66-.124) |
| **4** | White non-  British | .79(.52-1.21) | .85(.56-1.29) | 3.21(1.5-  6.80)** | 3.32(1.55-  7.12)** | 1.10(.82-  1.46) | 1.09(.82-  1.45) |
|  | mixed | .88(.48-1.59) | .82(.45-1.48) | 1.94(.713-5.27) | 2.14(.77-5.94) | 1.24(.85-  1.83) | 1.26(.85-  1.86) |
|  | Asian/south  Asian | 1.22(.76-1.96) | 1.14(.71-1.82) | 3.39(1.43-  8.021)** | 3.89(1.62-  9.36)** | 1.04(.75-  1.44) | 1.05(.76-  1.46) |
|  | Black African | 1.07(.56-2.07) | .98(.510-1.87) | 4.98(1.35-  18.31)* | 4.31(1.15-  16.12) * | 1.86(1.29-  2.68)** | 1.82(1.26-  2.62)** |
|  | Black  Caribbean | .72(.43-1.21) | .73(.44-1.22) | 5.16(1.86-  14.29)** | 5.77(2.06-  16.23)** | 1.48)1.09-  2.03)* | 1.49(1.09-  2.04)* |

|  | Black British | .75(.48-1.18) | .75(.48-1.17) | 4.07(1.75-  9.45)** | 3.78(1.61-  8.85)** | 1.66(1.28-  2.17)*** | 1.65(1.26-  2.15)*** |
| --- | --- | --- | --- | --- | --- | --- | --- |
|  | Other | IRR = .64, CI  = .51-  .75,.095(0.22-  .39)** | .08(0.20-.34)** |  |  | 0.60(.18-  2.01) | .63(.19-2.08) |
| **5** | White Non-  British | .71(.31-1.61) | .89(.40-1.99) | 2.9(.66-12.65) | 2.82(.61-12.90) | 1.27(.74-  2.18) | 1.25(.73-  2.15) |
|  | mixed | .80(.29-2.23) | 1.02(.37-2.76) |  |  | 1.59(.87-  2.93) | 1.68(.91-  3.09) |
|  | Asian/south  Asian | .65(.37-1.14) | .77(.44-1.33) | 2.73(1.0-  7.50)** | 3.17(1.11-  9.04)** | 1.37(.95-  1.97) | 1.40(.97-  2.02) |
|  | Black African | .35(.07-1.74) | .25(.05-1.18) |  |  | .72(.18-  2.92) | .68(.17-2.76) |
|  | Black  Caribbean | .589(.26-1.33) | 1.04(.52-2.10) | 1.74(.42-7.26) | 1.43(.32-6.28) | 1.09(.61-  1.96) | 1.10(.61-  1.98) |
|  | Black British | 1.32(.65-2.67) | 1.05(.52-2.10) | 7.83(1.63-  37.68)** | 6.19(1.24-  30.98)* | 2.11(1.45-  3.07)*** | 2.01(1.37-  2.97)**** |
|  | Other | .30(.08-1.13) | .23(.064-.85) |  |  | .72(.23-  2.27) | .70(.22-2.19) |
