# Supplementary material for: Understanding the intersections between ethnicity, area-level deprivation, and inpatient-related features amongst patients with psychotic disorders: a mental health electronic records analysis
Source: Soc Psychiatry Psychiatr Epidemiol. 2025 May 5;60(8):1957–69. doi: 10.1007/s00127-025-02908-1 (PMC12325388; doi:10.1007/s00127-025-02908-1)
Supplement: Supplementary file 2 — Supplementary Material 2 [file 127_2025_2908_MOESM2_ESM.docx]

**Title:** Understanding the intersections between ethnicity, area-level deprivation and inpatient-related features amongst patients with psychotic disorders: a mental health electronic records analysis

**Supplementary Material 2- Sensitivity analysis**

**Table S5a:** *Unadjusted and adjusted odds ratios of associations between deprivation and compulsory admission and admission to PICU, controlling for area-level clustering in only patients with a primary diagnosis of non-affective psychotic disorders (n=3,030)*

| Compulsory admission | | | PICU |  |
| --- | --- | --- | --- | --- |
| Deprivation  quintile | Unadjusted OR  Model 1 | Adjusted OR  Model 2 | Unadjusted OR  Model 1 | Adjusted OR  Model 2 |
| 4 | .53(.53-.53)*** | .53(.51-.54)**** | 1.23(1.23-1.23)*** | 1.33(1.29-1.37)*** |
| 3 | .61(.61-.61)*** | .59(.56-.61)*** | 2.33(2.33-2.33)*** | 2.58(2.52-2.65)*** |
| 2 | .70(.70-.70)*** | .66(.62-.70)*** | 2.58(2.58-2.58)*** | 2.73(2.67-2.79)*** |
| 1 Most deprived | .66(.66-.66)*** | .61(.58-.65)*** | 2.33(2.33-2.33)*** | 2.44(2.40-2.48)*** |

*<0.05, **<0.01, ***<0.001 Comparison group = quintile 5 (least deprived) Model 2: adjusted for age, gender, ethnicity

**Table S5b:** *Unadjusted and adjusted Incidence Rate Ratios of associations between deprivation and length of stay and number of admissions, controlling for area clustering in only patients with a primary diagnosis of non-affective psychotic disorders (n=3,030)*

|  | LOS |  | Number of admissions | |
| --- | --- | --- | --- | --- |
| Deprivation quintile | Unadjusted IRR  Model 1 | Adjusted IRR  Model 2 | Unadjusted IRR  Model 1 | Adjusted IRR  Model 2 |
| 4 | .86(.86-8.6)*** | .82(.76-.87)*** | 1.0(1.0-1.0)*** | 1.0(1.0-1.1)*** |
| 3 | .82(.82-.82)*** | .76(.71-.80)*** | 1.22(1.22-1.22)*** | 1.23(1.22-1.25)*** |
| 2 | .84(.84-.84)*** | .78(.74-.80)*** | 1.29(1.29-1.29)*** | 1.28(1.27-1.29)*** |
| 1Most deprived | .85(.85-.85)*** | .83(.80-.86)*** | 1.28(1.28-1.28)*** | 1.29(1.28-1.31)*** |

*<0.05, **<0.01, ***<0.001 Comparison group = quintile 5. Model 2: adjusted for age, gender, ethnicity

**Table S5c** *Unadjusted and adjusted odds ratios of associations between ethnicity and compulsory admission and admission to PICU (n=3,030)*

| Compulsory admission | | | Admission to PICU | |
| --- | --- | --- | --- | --- |
| Ethnicity | Unadjusted OR  Model 1 | Adjusted OR  Model 2 | Unadjusted OR  Model 1 | Adjusted OR  Model 2 |
| White non-British | .88(.61-1.27) | .86(.59-1.25) | 1.25(.83-1.88) | 1.05(.69-1.59) |
| Mixed | 1.55(.86-2.80) | 1.52(.84-2.74) | 2.24(1.40-3.58)** | 1.86(1.15-3.10)* |
| Asian | 1.49(.92-2.43) | 1.47(.90-2.40) | 1.08(.67-1.75) | 1.0 (.61-1.63) |
| Black African | 1.36(.94-1.99) | 1.39(.95-2.03) | 1.71(1.20-2.44)** | 1.84(1.28-2.64)** |
| Black Caribbean | 1.81(1.28-2.58)** | 1.78(1.24-2.54)** | 2.10(1.54-2.86)*** | 1.93(1.41-2.66)*** |
| Black British | 1.47 (1.08-2.00) | 1.42(1.04-1.94)* | 2.42(1.81-3.22)*** | 2.02(1.51-2.72)*** |
| Other | 1.62(.7*2-.363)* | 1.56(.69-3.5) | 1.01(.47-2.18) | .85(.38-1.87) |

*<0.05, **<0.01, ***<0.001. Comparison group = White British Model 2: adjusted for age, gender, deprivation IMD

**Table S5d:** *Unadjusted and adjusted Incidence Rate Ratios of associations between ethnicity and length of stay and number of admissions (n=3,030)*

|  | LOS |  | Number of admissions | |
| --- | --- | --- | --- | --- |
| Ethnicity | Unadjusted IRR  Model 1 | Adjusted IRR  Model 2 | Unadjusted IRR  Model 1 | Adjusted IRR Model  2 |
| White non-British | .79(.68-.93)* | .82*.70-.96)* | .92(.82-1.0) | .89(.79-1.0) |
| Mixed | 1.1(.86-1.34) | 1.1(.89-1.34) | 1.26(1.1-1.46)** | 1.22(1.06-1.40)** |
| Asian | .93(.78-1.11) | .97(.82-1.16) | .97(.95-1.10) | .95(.84-1.08) |
| Black African | 1.24(1.1-1.42)* | 1.2(1.05-1.39)* | 1.13(1.02-1.25)* | 1.13(1.02-1.25)* |
| Black Caribbean | .99(.95-1.20) | 1.01(.89-1.14) | 1.13(1.04-1.24)** | 1.10(1.01-1.20)* |
| Black British | 1.1(.95-1.2) | 1.1(.98-1.2) | 1.25(1.16-1.36)*** | 1.20(1.10-1.30)*** |
| Other | .58(.44-.7*7)**** | .63(.48-.83)** | .934(.76-1.15) | .90(.73-1.10) |

*<0.05, **<0.01, ***<0.001. Comparison group = White British. Model 2: adjusted for age, gender, deprivation IMD
